# Toxicity of *Beauveria bassiana*-28 Mycelial Extracts on Larvae of *Culex quinquefasciatus* Mosquito (Diptera: Culicidae)

**DOI:** 10.3390/ijerph15030440

**Published:** 2018-03-03

**Authors:** Perumal Vivekanandhan, Thangaraj Kavitha, Sengodan Karthi, Sengottayan Senthil-Nathan, Muthugoundar Subramanian Shivakumar

**Affiliations:** 1 Molecular Entomology Laboratory, Department of Biotechnology, School of Biosciences, Periyar University, Salem 636 011, Tamil Nadu, India; mosqvk@gmail.com (P.V.); kavithabio10@gmail.com (T.K.); skentomology@gmail.com (M.S.S.); 2 Division of Biopesticides and Environmental Toxicology, Sri Paramakalyani Centre for Excellence in Environmental Sciences, Manonmaniam Sundaranar University, Alwarkurichi, Tirunelveli 627 412, Tamil Nadu, India; karthientomology@gmail.com

**Keywords:** *Beauveria bassiana*-28, *Culex quinquefasciatus*, FT-IR, GC-MS, ethyl acetate, midgut, biopesticide

## Abstract

Microbial-based pest control is an attractive alternative to chemical insecticides. The present study sought to evaluate the toxicity of the entomopathogenic fungus *Beauveria bassiana*-28 ethyl acetate extracts on different larval stages and pupae of *Culex quinquefasciatus* mosquitoes. *B. bassiana*-28 ethyl acetate mycelial extracts produced mosquitocidal activity against larvae and pupae which was comparable to that of the commercial insecticide *B. bassiana*-22 extract. The LC_50_ (lethal concentration that kills 50% of the exposed larvae) values of *B. bassiana*-28 extracts for 1st to 4th instar larvae and pupae were 11.538, 6.953, 5.841, 3.581 and 9.041 mg/L respectively. Our results show that *B. bassiana*-28 ethyl acetate mycelial extract has strong insecticidal activity against larval and pupal stages of *Cx. quinquefasciatus*. Fourier transform infrared spectrum study of *B. bassiana*-28 extract shows peaks at 3226.91; 2927.94; 1593.13; 1404.18; 1224.18; 1247.94; 1078.21; 1018.41; 229.69; and 871.82 cm^−1^. Major spectral peaks were observed at 3226.91 cm^−1,^ assigned to N–H stretching, 2927.94 cm^−1^ assigned to C–H bonding and 1595.13 cm^−1^ assigned to C–O stretching. Gas Chromatography-Mass Spectrometry studies of *B. bassiana*-28 ethyl acetate crude extract showed presence of six major compounds viz. *N*-hexadecanoic acids (13.6040%); *Z,Z*-9,12 octadecadienic acid (33.74%); 9-eicosyne (10.832%); heptacosane (5.148%); tetrateracontane (5.801%); and 7 hexyleicosane (5.723%). Histology of mosquito midgut tissue shows tissue lysis as a result of *B.bassiana*-28 extract exposure. The study shows that bioactive molecules obtained from *B. bassiana*-28 mycelial extract has insecticidal properties and can be used as alternative for mosquito control.

## 1. Introduction

Mosquitoes are responsible for several vector-borne diseases [1]. Mosquitoes are classified into three subfamilies: *Anophelinae*, *Culicinae* and *Toxorhynchitinae* [2]. Female *Culex quinquefasciatus* mosquitoes are responsible for lymphatic filariasis in tropical and subtropical regions [3,4,5,6]. Human filariasis is a major public health hazard and remains a challenging socioeconomic problem in India and other tropical countries. Many researchers have reported that mosquitoes show resistance to synthetic chemical insecticides [7,8,9]. In this scenario, bioactive compounds of biological origin from bacteria, fungus, plants and entomopathogenic microbes are being bio-prospected as alternatives to chemical insecticides [10,11,12,13,14]. The advantages of these biopesticides are due to their biodegradability, target specificity, eco-friendly nature and their usefulness as tools to manage insecticide resistance in mosquitoes [15,16].

Several studies on entomopathogenic fungi (EPF) and their metabolites have shown their control potential in various stages of mosquitoes [17,18]. Secondary metabolites from *Chrysosporium* [19], *Metarhizium* and *Beauveria* [20,21]; *Culicinomyces* [22], *Verticillium* [19] and *Piper* [23] have been evaluated for their insecticidal potential on mosquitoes and houseflies [24]. Entomopathogenic fungus, particularly *B. bassiana*, are well known as insect pathogens against agricultural lepidopteran pests [25,26]. These EPF *Beauveria* spp., are highly specific to mosquitoes [27] and can be developed as potential insecticides for the control of mosquito larvae. *B. bassiana* is a cosmopolitan entomopathogenic fungus found in nature in soils. *B. bassiana*-derived insecticides have nowadays been registered and commercially developed worldwide as agricultural pest control methods [28]. *Beauveria bassiana* conidia, blastospores and their secondary metabolites are used for controlling the mosquito population at a laboratory level [29]. In the present study we investigated the toxicity of our isolate *B. bassiana*-28 extract in comparison with the commercially available microbial insecticide *Beauveria bassiana*-22 extract against different stages (Ist–IVth instar larvae) and pupae of *Cx. quinquefasciatus* mosquito and also performed larval histopathological studies at different time durations (6, 12 and 24 h post-treatment).

## 2. Materials and Methods 

### 2.1. Source of Culture

**Figure 1:**
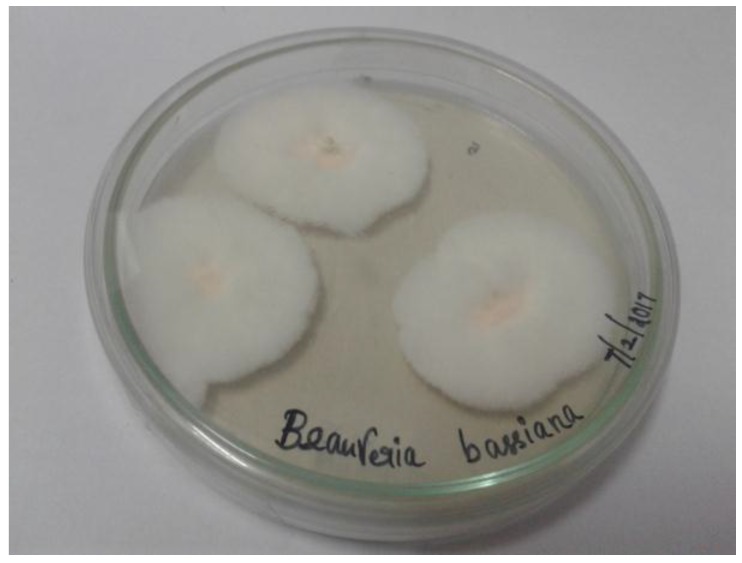
Seven days old strains of *B. bassiana*-28*.*

*B. bassiana*-28 fungal culture was isolated from dead cadavers of *Spodoptera litura* (tobacco cutworm or cotton leaf worm) insects in a cotton field in Dharmapuri, Tamil Nadu, India. *B. bassiana*-28 (Figure 1) was sub-cultured on potato dextrose agar (PDA) medium with added ampicillin (3 mg/100 mL) and incubated for 10 days at 25 °C ± 2 °C. *B. bassiana*-28 fungal culture was maintained and research work was carried out at the Molecular Entomology Laboratory (Salem, Tamil Nadu, India).

### 2.2. Commercial Microbial Insecticide B. bassiana-22

Commercially available powdered microbial insecticide *B. bassiana*-22 was procured from Manidharma Biotech, Pvt Ltd. (Chennai, Tamil Nadu, India). Commercial *Beauveria bassiana*-22 targets several insect pests such as root weevils, plant hoppers, Japanese beetle, black vine weevil, spittlebug and white grubs and lepidopteron pests. The procured *B. bassiana*-22 was cultured on PDA, supplemented with ampicillin (3 mg/100 mL) and incubated for 10 days at 25 °C ± 2 °C. 

### 2.3. Morphological Identification of B. bassiana-28

Morphological identification was carried out for isolated or unknown *B.bassiana*-28 fungal strains based on morphological characteristics such as colony colour, aerial mycelial structures, pigment production and conidia stained with lacto-phenol cotton blue and viewed under light microscope (Olympus-CH20i, Mumbai, India) at 400× magnification.

### 2.4. Mass Culturing of B. bassiana-28

*B. bassiana-*28 broth was prepared for the mass culture of fungal mycelia as per the modified method of [30]. Four 1000 mL conical flasks, each containing 500 mL of potato dextrose broth (PDB), (dextrose 40 g, peptone 10 g, deionized water 1000 mL), were sterilized at 15 psi for 30 min. The broths were supplemented with 30 mg ampicillin, which acts as a bacterial control agent. *B. bassiana*-28 fungal conidia (1 × 10^7^ per mL) were inoculated and grown in PDB. The flasks were incubated at 25 °C ± 2 °C for 25 days.

### 2.5. Crude Extraction from B. bassiana-28

Mass culturing of *B. bassiana*-28 and *B. bassiana*-22 was carried out in a 1000 mL Erlenmeyer flask containing 500 mL of PDB. The flasks were incubated under the optimized culture conditions (pH 7.0 at 27 °C) for 25 days. The fungal biomass was removed from the medium with help of Whatman No. 1 filter paper and washed more than five times with distilled water to remove the unwanted broth particles. Fungal biomass (100 g) was transferred to 500 mL glass beakers containing ethyl acetate (250 mL) which was mixed with the mycelium for cold extraction for 20 days at 25 °C ± 2 °C. After complete extraction the liquid portion was separated from the mycelium by filtering through Whatman No. 1 filter paper. Separated secondary metabolite ethyl acetate extracts were finally concentrated using a rotary vacuum evaporator (Superfit-R/150/11, Mumbai, India) at 45 °C.

### 2.6. Thin Layer Chromatography 

**Figure 2:**
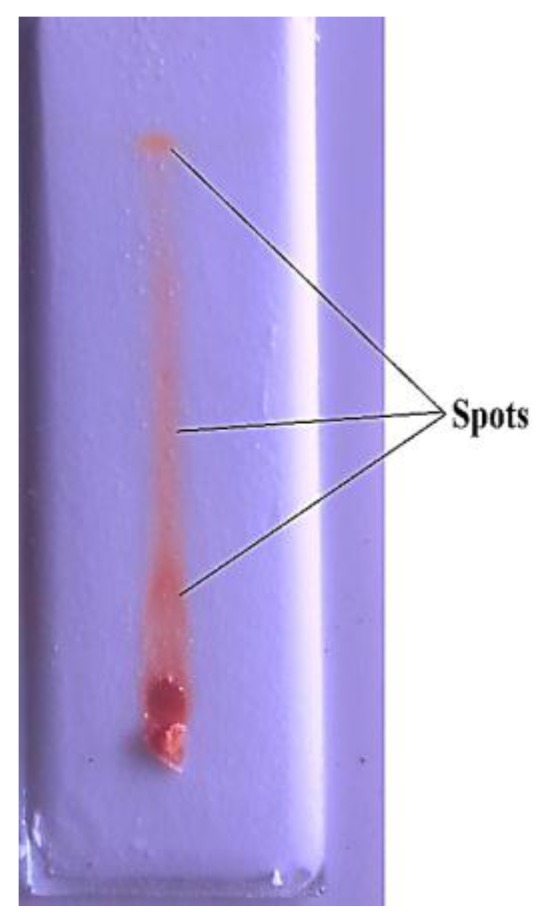
Thin layer chromatography of *B. bassiana*-28 extract. The mobile phases were chloroform: methanol in ratios of 10; 9:1; 8:2; 7:3; 6:4; 5:5; 4:6; 3:7; 2:8; 1:9; 10 and the retention factor (Rf) values of spots were 0.3333, 0.4444, 0.5555.

Thin layer chromatography **(**TLC) was performed on commercial silica gel-H TLC plates (chloride-0.02%, sulphate-0.02%, iron-0.02%, heavy metals-0.02% and pH-7) for principal components separation. The developed TLC plates were dried at room temperature. After air drying *B.bassiana*-28 extract were spotted at center of the plate with the help of a capillary tube. Then we prepared different solvent systems as mobile phases for thin layer chromatography because the biological molecules can be separated by different solvent system. The mobile phase solvent systems were chloroform:methanol in several ratios (10; 9:1; 8:2; 7:3; 6:4; 5:5; 4:6; 3:7; 2:8; 1:9; 10). After the running process the plates were observed under UV light at 350 nm. The retention factor (Rf) values were calculated using Equation (1) and based on the movement of samples in TLC plate (Figure 2).

(1) $$\mathrm{Rf}=\frac{\mathrm{Distance}\;\mathrm{travel}\;\mathrm{by}\;\mathrm{solute}}{\mathrm{Distance}\;\mathrm{travel}\;\mathrm{by}\;\mathrm{solvent}}$$

### 2.7. Mosquito Culture

*Cx. quinquefasciatus* egg rafts were obtained from the Institute of Vector Control Zoonoses, (IVCZ, Hosur, Tamil Nadu, India). The egg rafts were maintained in 2 L plastic jars containing tap water. The larvae were fed with dog biscuits and millet powder and yeast powder in 3:3:1 ratio. Larvae were kept at 27 °C ± 2 °C and 70–85% relative humidity with a 12:12 light and dark photoperiod.

### 2.8. Larval Bioassay

Larval mortality bioassays were carried out according to the method suggested by the World Health Organization (WHO) [31], with slight modifications. Extracted *B. bassiana*-28 and *B. bassiana*-22 mycelia extract were transferred individually to 250 mL round bottom flasks, then the ethyl acetate solvent was removed by using a rotary evaporator, (Superfit- R/150/01, Mumbai, India). The bioassay has five testing concentrations and each concentration had three replicates of twenty larvae each. Test containers containing 20 mosquito larvae were stored in 150 mL plastic cups containing 99 mL of distilled water with the desired concentration (i.e., 25, 50,100,150, 200 and 250 μg/mL). In the control 20 individuals were exposed to the same dose of dimethyl sulfoxide (DMSO) as negative control. After 24 h exposure, mortality (%) was calculated and corrected with control mortality using the Abbott formula [32]. The larval mortality was calculated after 24 h post treatment. LC_50_ and LC_90_ values were calculated by probit analysis using the SPSS-16.0 software (IBM-Corporation, Bengaluru, Karnataka, India).

(2) $$\mathrm{Percentage}\;\mathrm{of}\;\mathrm{mortality}=\frac{\mathrm{Number}\;\mathrm{of}\;\mathrm{dead}\;\mathrm{larvae}}{\mathrm{Number}\;\mathrm{of}\;\mathrm{larvae}\;\mathrm{introduced}}\;\times \;100$$

### 2.9. Pupal Toxicity Tests

*Cx quinquefasciatus* pupae from the laboratory maintained culture were used to examine the pupal toxicity of *B. bassiana*-28 and *B. bassiana*-22 extracts. Twenty pupae were transferred to 150 mL plastic containers containing 99 mL of distilled water. Five different concentrations of extracts (i.e., 25, 50, 100, 150, 200 and 250 μg/mL) were separately dissolved in DMSO (1 mL) and the dissolved fungal extracts were added to the water in the bioassay vessels. Each concentration had three replicates and each replicate had twenty pupae. Mortality was calculated 24 h post treatment, and mortality in the treatments and control was corrected using Abbott’s formula [32]. The LC_50_ and LC_90_ values were calculated from the toxicity data by probit analysis using SPSS-16.0 software.

## 3. Gas Chromatography-Mass Spectrophotometer (GC-MS) Analysis

A Clarus 680 30 m × 0.25 mm ID × 250 μm silica column was used for GC analysis of the chemical constituents. This column was packed with elite-5MS (5% biphenyl 95% dimethylpolysiloxane). The chemical constituents were separated by using He at a constant flow of 1 mL/min as a carrier gas. The crude extracts (1 μL) were injected to the GC-MS instrument at 260 °C during the column running time. The temperature ramp was as follows: 60 °C (2 min); followed by 300 °C at the rate of 10 °C min^−1^; and finally 300 °C, where it was held for 6 min. The mass detector conditions were 240 °C; ion source temperature at 240 °C; and ionization mode electron impact at 70 eV, a scan time 0.2 s and scan interval of 0.1 s. The fragments from 40 to 600 Da were collected. 

### Histological Studies

The 6, 12 and 24 h post-treated and control 4th instar *Cx. quinquefasciatus* larvae were fixed in 3% formaldehyde solution for 2 h at 4 °C. The blocks were cooled at 27 °C for 3 h and cut to 8 μm thickness 1.3 mm ribbons with a microtome (Berlin, Germany). Cross-sectioned larval gut was stained with haematoxylin and eosin stain. After air drying sections was viewed under a light microscope (Olympus-CH20i) at a magnification of 400×.

## 4. Results

### 4.1. Larval Bioassay

Based on LC_50_ and LC_90_ values it was found that *B. bassiana*-28 mycelium extract had insecticidal activity similar to that of the commercial microbial insecticide *B. bassiana*-22 on 1st to 4th instar of *Cx. quinquefasciatus* (Table 1 and Table 2).

### 4.2. Thin Layer Chromatography 

Thin Layer Chromatography was analysed. *B. bassiana*-28 extract showed three spots with Rf values of 0.3333, 0.4444, and 0.5555, respectively (Figure 2).

### 4.3. Gas Chromatography-Mass Spectrometry Analysis of B. bassiana-28 Ethyl Acetate Mycelial Extract

**Figure 3:**
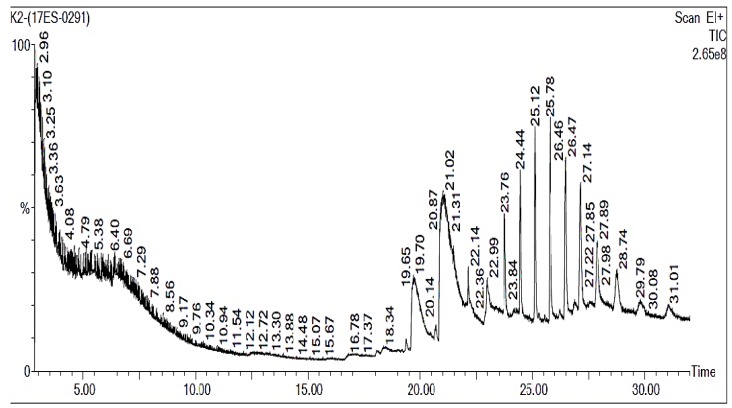
Compounds were identified from *B.bassiana*-28 extracts. Oven initial temp 60 °C for 2 min, ramp 10 °C min^−1^ to 300 °C, hold 6 min, Inject auto = 250 °C, volume = 1 μL, split = 10:1, carrier gas = He, solvent delay = 2.00 min, transfer temp = 240 °C, source temp = 240 °C, scan 50 to 600 Da, column 300 m~250 μm.

Gas Chromatography-Mass Spectrometry results obtained from the *B. bassiana*-28 indicated the presence of several major compounds viz. *N*-hexadecanoic acid (19.695%), eicosanoic acid (21.016%), octadecanoic acid (21.466%), tridecanoic acid (22.081%), pentadecanoic acid (22.136%), tetradecanoic acid (22.986%), octadecanoic acid (23.757%), eicosanoic acid (24.442%), heptadecanoic acid (25.117%), tridecanoic acid (25.778%), octadecanoic acid (26.468%), tridecanoic acid (27.143%), dodecanoic acid (27.888%), l-(+)-ascorbic acid 2,6-dihexadecanoate (28.749%) (Figure 3 and Table 3). Six major compounds—*N*-hexadecanoic acid (13.6040%), *Z,Z*-9,12 dectadecadienoic acid (33.74%), 9-eicosyne (10.832%), heptacosane (5.148%), tetrateracontane (5.801%); and 7 hexyleicosane (5.723%)—may be involved in the insecticidal activity.

### 4.4. Fourier transform infrared spectrum Analysis of B. bassiana-28 Ethyl Acetate Mycelial Extract 

**Figure 4:**
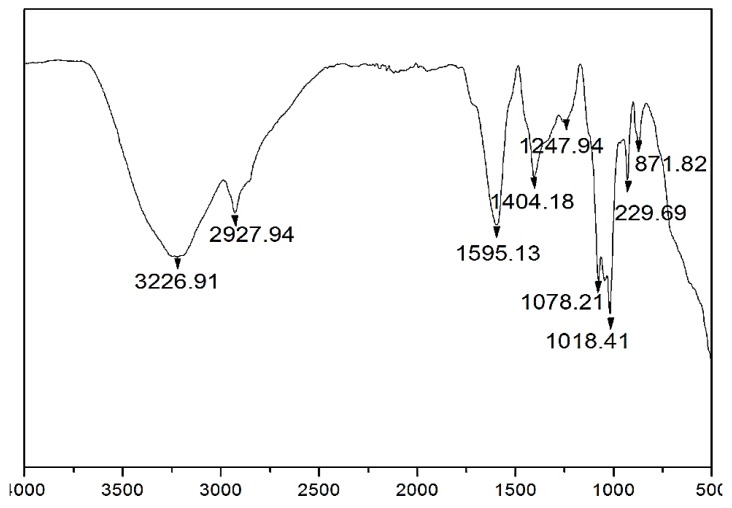
Fourier transform infrared (FT-IR) spectrum analysis of *B. bassiana*-28 extract, scanning it in the range 500–4000 cm^−1^ at a resolution value of 4 cm^−1^.

The intracellular ethyl acetate metabolites were illustrated using Fourier transform infrared (FT-IR) analysis (Figure 4; Table 4). The results indicate the presence of bands with peak values at 3226.91; 2927.94; 2858.51; 1595.13; 1404.18; 1247.94; 1078.21; 1018.41; 929.69; 871.82 and 503.42 cm^−1^ (Table 4). *B. bassiana* -28 extracts have prominent peaks in the FT-IR spectra at 3226.91 cm^−1^ corresponding to N–H stretching vibrations. The strong band at 1595.13 cm^−1^ is assigned to C=O stretching. The two bands at 1078.21 cm^−1^, S=O stretching and 1018.41 cm^−1^, C–O stretching, are strong support to the presence of aliphatic and aromatic amines, respectively.

### 4.5. Histological Studies of Larvae of Cx. quinquefasciatus 

**Figure 5:**
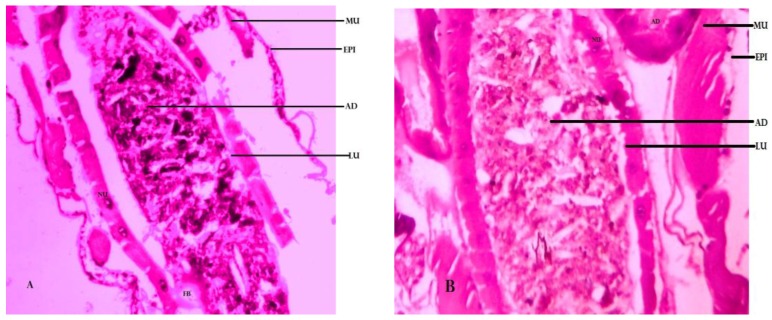

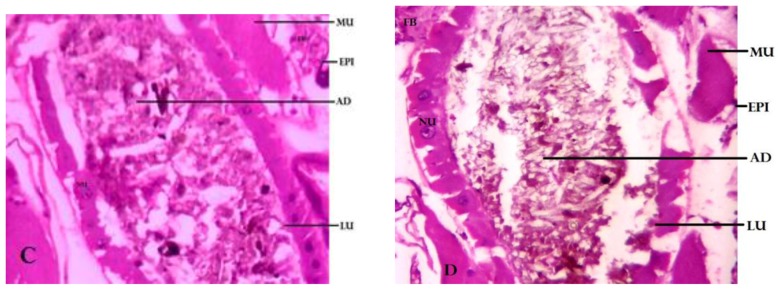
Cross section of *Cx. quinquefasciatus* larvae untreated and treated with *B.bassiana*-28 extract Control (**A**) (untreated) compared with (**B**) 6 h Treated (**C**) 12 h treated and (**D**) 24 h treated, Vacuolated gut epithelium (epi), gut lumen (lu), adipose tissue(ad), muscles (mu), nucleus (nu) and fat body (fb). Larval mid-gut section was stained with haematoxylin and eosin and stained mid-gut tissues viewed and photographed under light microscope at 400× magnification.

Histopathological results clearly show that midgut epithelial cells (epi) were severely damaged by *B. bassiana*-28 extract. Gut tissue of lumen was mutually by way of a thin peritrophic membrane (Pm) in 6 h treatment as compared to control (Figure 5B), whereas, at 12 h and 24 h treated midgut epithelium layer was damaged and cells were vacuolated but remained together with the nuclei and membrane was entirely bust during action with *B. bassiana*-28 (Figure 5B,C) also the lumen substance (lu) seeped out into muscles cells (mu). In muscles was appeared barely injured and fat bodies were confused.

## 5. Discussion

In the present study, *B. bassiana*-28 and commercial *B. bassiana*-22 were found to produce toxicity in all stages like larval and pupal *Cx. quinquefasciatus*. Result clearly shows that *B. bassiana*-28 extract has strong mosquitocidal activity against 1st to 4th instar larvae of *Cx. quinquefasciatus,* (Table 1 and Table 2). Other fungi of the same species to which *Beauveria* belongs are sources of bioactive metabolites and conidia that have mosquitocidal properties under field and laboratory conditions [21,33,34]. Clark et al. [35] reported that *Beauveria bassiana* shows remarkable pathogenicity on three mosquito species such as *Anopheles*, *Aedes* and *Culex*. The entomopathogenic fungal pathogen *Beauveria bassiana* fungal conidia and blastospores reduced the malarial vector *Anopheles stephensi* survival rates [36].

*B. bassiana*-22 extracts toxicity has been proved with highest toxicity against the *Cx. quinquefasciatus* secondary metabolites from other reports on the same species. *Beauveria bassiana* show remarkable insecticidal properties against insecticide resistant and susceptible *Anopheles arabiensis* mosquitoes at different temperatures [37]. The FT-IR spectrum of the secondary metabolites of *B. bassiana*-28 contains important peaks indicating the presence of N–H stretching; C–H bending; C–H stretching; C=C bending; C=O stretching; C–H bending; S=O stretching; C–O stretching; N–O stretching; C=C bending; C–B stretching groups. Ethyl acetate mycelium extract showed the presence of prominent functional groups these are maybe involved in the mosquitocidal activity. GC-MS analysis marks exhibited important mosquitocidal compounds potentially responsible for insecticidal activity, namely, *N*-hexadecanoic acid (13.6040%) [38]; *Z, Z*-9,12 dectadecadienoic acid (33.74%); 9-eicosyne (10.832%); heptacosane (5.148%); tetrateracontane (5.801%); and 7-hexyleicosane (5.723%) are also thought to be involved in insecticidal activity. In our results show the major compound in assessment with the standard was *N*-hexadecanoic acid, and based on the above we infer that *N*-hexadecanoic acid from *B. bassiana*-28 mycelial extract may be the principal metabolite which confers insecticidal activity to the extract.

## 6. Conclusions

*B. bassiana*-28 mycelial extracts produced toxicity in larvae and pupae of *Cx quinquefasciatus.* In addition these extract produce considerable tissue damage to the midgut of mosquito larvae. Characterization of extracts showed that *N*-hexadecanoic acid is the main constituent of the extract which has insecticidal properties. In future work on control of mosquito larvae, *N*-hexadecanoic acid can be used as an alternative to chemical insecticides.

## Figures and Tables

**Table 1:** Larvicidal and pupicidal activity of *B. bassiana*-28 fungal mycelium extract (ethyl acetate) against larvae and pupa of *Cx. quinquefasciatus* (after 24 h of exposure).

Mosquito Species	Larval Stages	Concentration (mg/L)	Mortality (%) ± S.D.	LC_50_ (LCL-UCL) mg/L	LC_90_ (LCL-UCL) mg/L	χ^2^ (df) 3
*Cx. quinquefasciatus*	1st Instar	Control	2.5 ± 0.12	11.538 (4.061–20.308)	16.155 (6.575–26.375)	4.276
		25	15.24 ± 0.8			
		50	43.33 ± 1.0			
		100	53.33 ± 1.0			
		150	71.66 ± 2.5			
		200	83.33 ± 1.5			
		250	91.66 ± 0.5			
	2nd Instar	Control	2.1 ± 0.11	6.953 (1.158–15.718)	10.790 (2.345–21.689)	3.089
		25	17.12 ± 1.0			
		50	43.33 ± 2.5			
		100	53.33 ± 1.0			
		150	61.66 ± 1.0			
		200	78.33 ± 2.0			
		250	83.33 ± 1.0			
	3rd Instar	Control	1.8 ± 0.10	5.841 (1.151–12.787)	8.337 (1.993–16.673)	2.978
		25	21.45 ± 1.2			
		50	63.33 ± 0.5			
		100	73.33 ± 0.5			
		150	83.33 ± 1.5			
		200	93.33 ± 2.5			
		250	96.66 ± 0.5			
	4th Instar	Control	2.1 ± 0.18	3.581 (2.254–18.730)	5.265 (3.437–23.043)	3.421
		25	37.23 ± 1.3			
		50	71.66 ± 1.5			
		100	78.33 ± 2.0			
		150	86.66 ± 1.0			
		200	93.33 ± 0.5			
		250	100.00 ± 1.5			
	Pupa	Control	2.7 ± 0.19	9.041 (2.975–16.369)	12.104 (4.532–20.504)	3.404
		25	25.42 ± 1.2			
		50	61.66 ± 1.5			
		100	78.33 ± 3.0			
		150	86.66 ± 1.0			
		200	93.33 ± 0.5			
		250	100.00 ± 0.0			

LC_50_: lethal concentration that kills 50% of the exposed larvae and pupa LC_90_: lethal concentration that kills 90% of the exposed larvae and pupa; UCL: upper confidence limit (95% fiducial limit); LCL: lower confidence limit (95% fiducial limit); χ^2^: chi-square; df: degrees of freedom; S.D.: standard deviation.

**Table 2:** Larvicidal and pupicidal activity of commercial insecticide *B. bassiana*-22 fungal mycelium extract (ethyl acetate) against larvae and pupa of *Cx. quinquefasciatus* (after 24 h of exposure).

Mosquito Species	Larval Stages	Concentration (mg/L)	Mortality (%) ± S.D.	LC_50_ (LCL-UCL) mg/L	LC_90_ (LCL-UCL) mg/L	χ^2^ (df) 3
*Cx. quinquefasciatus*	1st Instar	Control	2.5 ± 0.10	10.523 (3.237–19.576)	15.843 (5.871–26.807)	0.774
		25	18.02 ± 0.9			
		50	41.66 ± 1.5			
		100	60.00 ± 1.5			
		150	78.33 ± 1.0			
		200	88.33 ± 0.5			
		250	98.33 ± 0.5			
	2nd Instar	Control	1.5 ± 0.00	6.840 (1.819–16.649)	11.792 (2.069–24.581)	0.721
		25	26.11 ± 0.8			
		50	36.66 ± 2.0			
		100	58.33 ± 1.5			
		150	63.33 ± 1.0			
		200	71.66 ± 1.0			
		250	83.33 ± 2.5			
	3rd Instar	Control	2.0 ± 0.18	4.616 (1.010–11.781)	7.631 (1.293–16.992)	0.704
		25	28.32 ± 1.0			
		50	55.00 ± 0.5			
		100	73.33 ± 0.5			
		150	81.66 ± 1.0			
		200	91.66 ± 2.5			
		250	98.33 ± 1.0			
	4th Instar	Control	2.3 ± 0.0	2.674 (1.343–8.466)	4.605 (2.068–12.449)	0.036
		25	34.12 ± 1.0			
		50	61.66 ± 1.5			
		100	81.66 ± 0.5			
		150	91.66 ± 1.0			
		200	96.66 ± 0.5			
		250	100.00 ± 0.			
	Pupa	Control	0.00	8.364 (1.643–17.906)	13.552 (3.516–25.583)	0.465
		25	26.09 ± 0.7			
		50	40.00 ± 0.5			
		100	55.00 ± 0.5			
		150	70.00 ± 2.5			
		200	80.00 ± 1.0			
		250	88.33 ± 0.5			

**Table 3:** Major bioactive compounds identified in the ethyl acetate mycelium extracts of *B. bassiana*-28 using Gas Chromatography-Mass Spectrometry analysis.

S. No.	Retention time (min)	Compound Name	Molecular Weight	Formula	Area (%)	Biological Activity
1	16.695	*N*-Hexadecanoic Acid	256	C_16_H_32_O_2_	13.640	Pesticidal activity
2	21.016	(Z,Z)-9,12 Octadecadienoic Acid	280	C_18_H_32_O_2_	33.747	Anti-inflammatory activity
3	21.466	9-Eicosyne	278	C_20_H_38_	10.832	No activity reported
4	22.081	*cis*-9,10-Epoxyoctadecan-1-ol	280	C_18_H_32_O_2_	1.352	No activity reported
5	22.136	*N*-[Bromo-*N*-butyl]-2-piperidinone	233	C_9_H_16_ONBr	2.612	No activity reported
6	22.986	Hexatriacontane	506	C_36_H_74_	2.133	No activity reported
7	23.757	1-Bromo-2-methyldecane	234	C_11_H_23_Br	3.121	No activity reported
8	24.442	Tritetracontane	604	C_43_H_88_	3.647	No activity reported
9	25.117	Heptacosane	380	C_27_H_56_	5.148	Antibacterial
10	25.778	Tritetracontane	618	C_44_H_90_	5.801	Anti-bacterial, Anti-fungal
11	26.468	7-Hexyleicosane	366	C_26_H_54_	5.723	No activity reported
12	27.143	Nonacosane	408	C_29_H_60_	5.408	Anti-bacteria,
13	27.888	1-Chloroheptacosane	414	C_27_H_55_CI	3.866	No activity reported
14	28.749	9-Octyleicosane	394	C_28_H_58_	2.971	No activity reported

**Table 4:** *Beauveria bassiana*-28 mycelial crude metabolites of FT-IR Spectrum study.

Observed Wave Numbers (cm^−1^)	Peak Assignment	Visible Intensity	Functional Group
3226.91	N–H stretching	Broad shape	Aliphatic
2927.94	C–H bending	Medium	Alkane
2858.51	C–H stretching	Medium	Alkane
1595.13	C=O Stretching	Medium	Alkane
1404.18	C–H bending	Medium	Alkane
1247.94	C–O Stretching	Medium	Alkane, Ether
1078.21	S=O Stretching	Sharp	Sulfone
1018.41	C–O Stretching	Sharp	Alkane
929.69	N–O Stretching	Sharp	Aliphatic
871.82	C=C bending	Sharp	Alkane
503.42	C–Br Stretching	Medium	Alkane
